# Use of Photovoice to engage stakeholders in planning for patient-centered outcomes research

**DOI:** 10.1186/s40900-019-0166-y

**Published:** 2019-11-28

**Authors:** Jill D. Nault Connors, Marshall J. Conley, Laura S. Lorenz

**Affiliations:** 1 Department of Emergency Medicine, Indiana University School of Medicine, Fifth Third Bank Building, Third Floor, 720 Eskenazi Avenue, Indianapolis, IN 46202 USA; 2 Pipeline-to-Proposal Award to the Trustees of Indiana University, Bloomington, IN USA; 3 Visiting Scholar, Heller School for Social Policy and Management, Brandeis University, Waltham, MA USA

**Keywords:** Photovoice, Anxiety, Engagement, Patient-centered outcomes research

## Abstract

**Background:**

Research is needed to inform patient and provider decisions about how to best care for patients who go to the emergency department with complaints of chest pain when their symptoms are due to anxiety rather than a heart problem. However, this research may not be a high priority due, in part, to a lack of awareness for the severity of anxiety symptoms and the impact of anxiety on peoples’ daily lives. In this commentary article, we highlight the use of Photovoice as a unique method to share patients’ lived experience of anxiety with providers, researchers, and health system leaders.

**Main text:**

A brief background on Photovoice methods, the process of patient partner involvement in Photovoice, and the project’s Photovoice results (posters, photos and captions) is presented.

**Conclusion:**

Photovoice achieved its intended effects of increasing awareness of all stakeholders about the burden of anxiety in patients’ lives and the imperative of improving emergency department care for anxiety. This resulted in increased participation in a multi-stakeholder research partnership, critical health system support that included costs to the health system associated with implementing interventions to be tested, and submission of a patient-centered outcomes research proposal that is currently under review. In addition, Photovoice had positive benefits for participants including a therapeutic effect, may have increased group cohesion, and empowerment of patients as partners in the research process.

## Plain English summary

People with conditions like anxiety may know a lot about what it is like to live day to day with anxiety, but some of that knowledge will be hard to put into words in a way that others can understand it. Photovoice is a method which can help people share that tacit knowledge with others. Photovoice involves using a camera to take photos that match a thought or feeling about anxiety and develop a photo caption to share meaning of how the photo relates to what they’re going through. In this project, people with anxiety used Photovoice to get anxiety seen as something serious that needs to be treated just as you would treat a physical health problem. We [Photovoice participants] were motivated by a desire to improve care for everyone that goes to the emergency department due to fear of having a heart attack, and are sent home with no treatment or help when anxiety was the actual cause of our symptoms. Coming back to the ED for false alarms of heart attack wastes time and money that could be used to get patients the help they truly need the first time around. Photovoice added a face to the numbers of people this happens to, and worked to get people to talk about it and figure out a solution. Because they trusted us and saw where we were coming from, our opinions about things like what treatments should be involved [in a research study] were important. Being part of the solution and not the problem is good. Feeling like you’re not the only one who goes through this is good.

## Introduction

This article highlights our unique use of Photovoice methods to engage stakeholders in patient-centered outcomes research (PCOR) focused on informing treatment options for persons with anxiety disorders seeking care in the Emergency Department setting. As defined by the Patient-Centered Outcomes Research Institute, PCOR “helps people and their caregivers communicate and make informed healthcare decisions, allowing their voices to be heard in assessing the value of healthcare options” [1]. During the planning phase of the research, a small group of patients, providers, and researchers met monthly over a six-month period to discuss patients’ experiences of health and healthcare related to anxiety. These discussions informed a comprehensive view of the problem and needed steps toward a solution [2]. In brief, symptoms of an anxiety disorder can be perceived by patients as a heart attack or other serious threat to health and cause patients to seek care in the ED. However, after tests have ruled out a heart attack or other serious event, patients are commonly discharged without discussion of anxiety as a potential underlying cause of symptoms or a referral for further evaluation and treatment of anxiety. Greater awareness of anxiety and prioritization of the research topic was needed, particularly among emergency medicine providers who could not be expected to appreciate the functional impairments of anxiety on work, family and social life during brief emergency department encounters. Hence, we initiated the Photovoice project to increase awareness of anxiety by documenting the lived experience of anxiety disorders and their impact on peoples’ lives and ensure that voices of patients were heard and valued. A second purpose was to create an opportunity for discussions about health and healthcare related to anxiety by disseminating Photovoice results among the growing multi-stakeholder research partnership.

This paper will provide a brief background on Photovoice, describe our process of patient-partner involvement in Photovoice, share the project’s Photovoice results (posters, photos and captions), and discuss the impact on stakeholders and stakeholder engagement in research. Engagement activities described in this paper were funded under a Pipeline to Proposal (P2P) Award by the Patient-Centered Outcomes Research Institute®. The overall purposes of the P2P award were to develop a multi-stakeholder research partnership, build capacity for patient-centered outcomes research, and inform a subsequent research application. In addition, the P2P award specifically prohibited use of funds for research purposes. Engagement activities, including the Photovoice project, performed under the P2P award were deemed non-human subjects research by our institutional review board. Toward that end, the results of the Photovoice project should not be interpreted as research.

## Main text

### Photovoice background

**Fig. 1:**
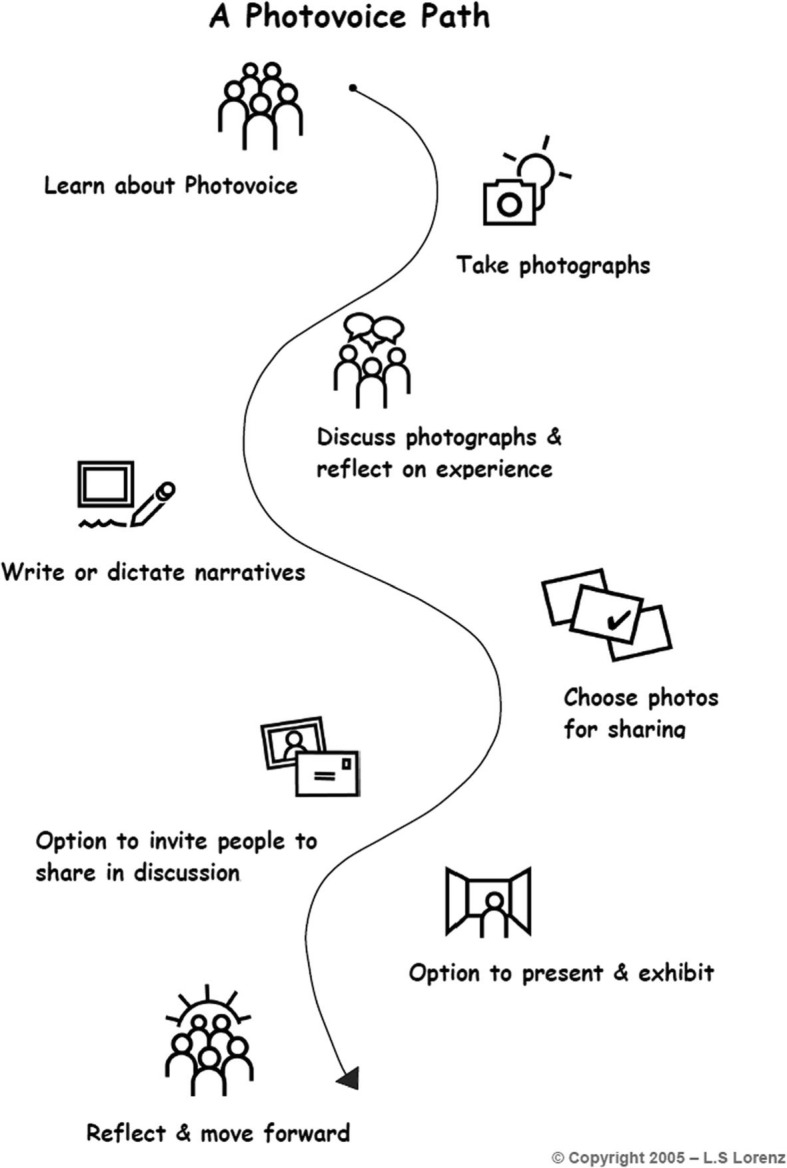
A Photovoice Path. Legend: The Photovoice process often involves a group of participants and facilitators working together to represent lives and point of view using photographs and captions (a.k.a. narratives) [8, 18, 25]. The steps of Photovoice as shown in Fig. 1, A Photovoice Path, involve identifying a topic to investigate with cameras; learning about the responsibilities of being a visual researcher; taking photos to represent experiences, thoughts, and feelings; discussing the photos together in a group; selecting some for exhibit and writing captions for them; and developing an exhibit to inform policymakers, communities, clinicians, and peers and encourage policy change [18]

Sharing information through the use of visuals (particularly photography) has been used in diverse fields such as public health, medicine, anthropology, sociology, education, and psychology to promote social justice and understanding of community experience for more than 50 years ([3]; [4–6];). Photovoice methods used today are based on the foundational work in participatory action research of Caroline C. Wang and colleagues in the 1990s [6–9]. Within this context, Photovoice asks people to represent their lives, point of view and experience using photographs and narratives (Fig. 1).

Photovoice has been applied to a wide variety of settings, people, and issues ranging from development needs of women in rural China [8] to community re-integration after deployment (add NOLA reference), and the experience of homelessness [10]. Researchers have also used Photovoice to create awareness of lived experience with chronic conditions and disabilities such as acquired brain injury [11], asthma [12], HIV/AIDS [13], mental illness [14], spinal cord injury [15], substance use disorders [16], and tuberculosis [17]. Applied to health and healthcare, Photovoice can help to illuminate gaps in clinical knowledge, contribute to efforts to improve quality and efficiency of care, and identify patient-centered goals and possibilities for healing [18]. The process of talking about participants’ photographs can help to bridge the discipline gap between patients and clinicians, bring out tacit knowledge and understanding, and generate common understanding of issues, contexts and solutions [19].

Individuals who participated in the Photovoice project were recruited as patient partners from initial listening groups conducted with adult patients who had lived experience of receiving care in the ED for low-risk chest pain and anxiety. The term patient partner specifically applies to patients engaged in the planning, conduct, or dissemination of research and should not be confused with the role of patient as a research subject. Six patient partners (AP, AB, CM, LS, MC, VC) along with the P2P Project Lead (JNC) and an expert Photovoice consultant (LL) embarked on a six-week Photovoice project to document the impact of anxiety on peoples’ lives. The patient partners who chose to participate were diverse in terms of age, gender, race, and occupation. All patient partners screened positively for high levels of anxiety on the Generalized Anxiety Disorder 7-item scale (GAD-7). For the purposes of engagement activities, self-report of anxiety was not confirmed as the GAD-7 is aligned with diagnostic criteria for generalized anxiety disorder and also has good operating characteristics for panic disorder, social anxiety disorder, and post-traumatic stress disorder [20].

Initial learning about Photovoice among participants included an introduction to the foundations and method of Photovoice, a review of ethics and safety guidelines, brief instruction on photo-taking tips, and practice developing captions based on sample photos. Participants were instructed to ask permission before taking someone’s photo, and to provide written consent for themselves and for any individuals appearing in photos to include the photos in the project exhibit. Participants then used disposable cameras for weekly photo assignments intended to describe both negative and positive aspects of lived experience with anxiety. Examples of guiding questions for weekly photo-taking included: ‘What is it like to live with an anxiety disorder?’ and ‘How does anxiety affect your health … work, family and social life?’ Each week, participants turned in their disposable cameras in for developing and reviewed printed copies of their photos from the prior week.

Patient partners approached their photo-taking seriously. They seemed to feel a responsibility to share their lived experience and contribute to improving quality of care for anxiety disorder in the emergency department. As a patient-partner peer leader noted:

We are trying to get anxiety seen as something serious, which it is. Anxiety is not just feeling anxious. It is something that can be debilitating. It needs to be taken more seriously in the Emergency Department. This project is our chance to talk about anxiety, so people can see where we are coming from. (Patient Partner Peer Leader).

As they shared their photos with their patient partner peers, the group used reflection questions based on the SHOWeD method to structure one-to-one and group discussions about the photos as well as their meaning and significance [9]. SHOWeD is an acronym for structured reflection questions [9]: What do you See here? What is really **H**appening here? How does this relate to **O**ur lives? Why does this situation, concern, or strength **e**xist? What can we **D**o about it?

Patient partners used journals to record their thoughts and support group discussions and caption-writing. Participants chose which of their photos to discuss with the group and share outside the project setting. Facilitators transcribed the focus group discussions and developed draft captions, using wording from the transcripts, for each photo chosen for the project exhibit by participants. Through an iterative process that involved taking additional photos, writing, reviewing, and discussion, participants refined their photo selections and what they wanted to say about them. During time outside of regular meetings, participants used a secure online platform (e.g., Slack) to communicate with each other and facilitators about their photos and captions, create a title for their exhibit, and develop a common format for their exhibit posters.

A wrap-up event brought together research stakeholders (patient partners, clinicians, researchers, health system leaders) to share learning from the P2P project including challenges to current care delivery in the ED relative to low-risk chest pain and anxiety, epidemiology of the problem (i.e., prevalence of low-risk chest pain and anxiety), a gallery exhibit of the Photovoice posters, semi-structured small group conversations among clinicians, investigators, and patient-partners, and opportunities to inform decision-making about treatment options through PCOR. Bringing together stakeholders at the event was intended to foster conversations about patient experiences with anxiety disorders as illustrated in the gallery exhibit, gain stakeholder input and feedback on the proposed research idea and question, gain support for prioritization of the research topic, and plan next steps for informing a research project.

## Photovoice results

**Fig. 2:**
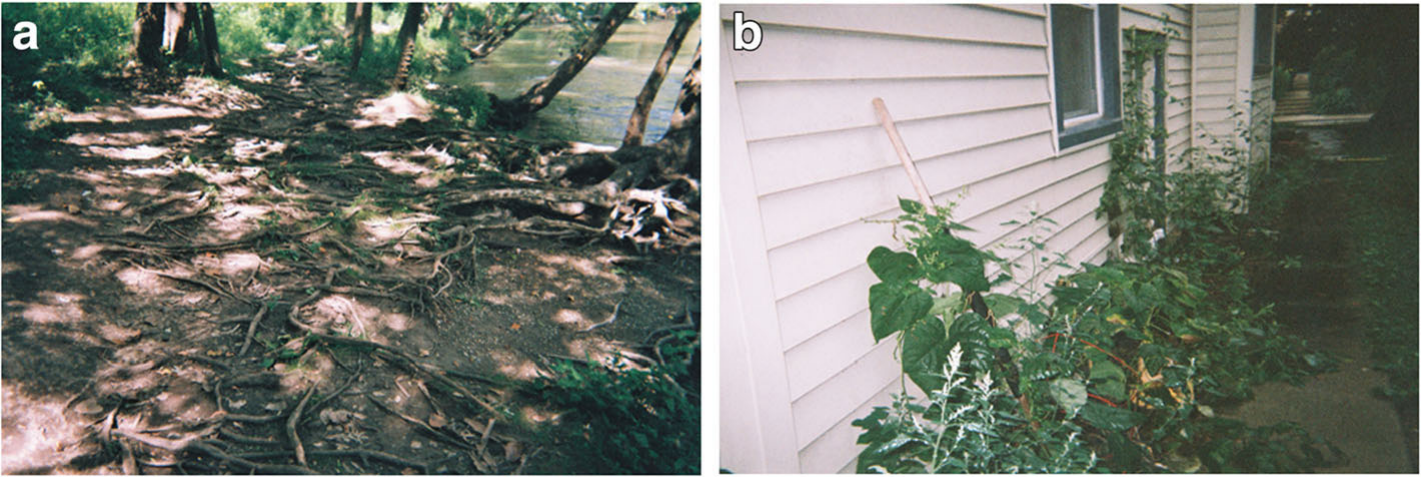
**a** and **b**: Anxiety for Marshall C. Legend 2**a**: This tree is beautiful, and you can see where it is going, until it goes in the dirt. And then you have no idea where it goes after that. And I kind of felt, that’s how my mind works. I like my thoughts, I like the way I think, I like the way I do things, but when I have anxiety, that’s when the roots become that sort of tangled mess and everything is sort of scary, and I don’t know where things are going, but I just have to remember at the end of the day, it still all leads back to the tree, it all leads back to me. I am not my anxiety. It is just something that happens. I looked at that tree, and I felt like things are going to be okay, no matter how bad things get, I am still me, and that is not going to change. “*I am not my anxiety”.* Legend 2**b**: I can feel the anxiety quickly creeping up. From my legs all the way to my head. I can feel it quickly restricting me. I can’t move no matter how much I struggle and being so tightly restrained makes me feel even more anxious and I can feel the vines grow tighter and tighter. “*Being so tightly restrained makes me feel even more anxious”*

**Fig. 3:**
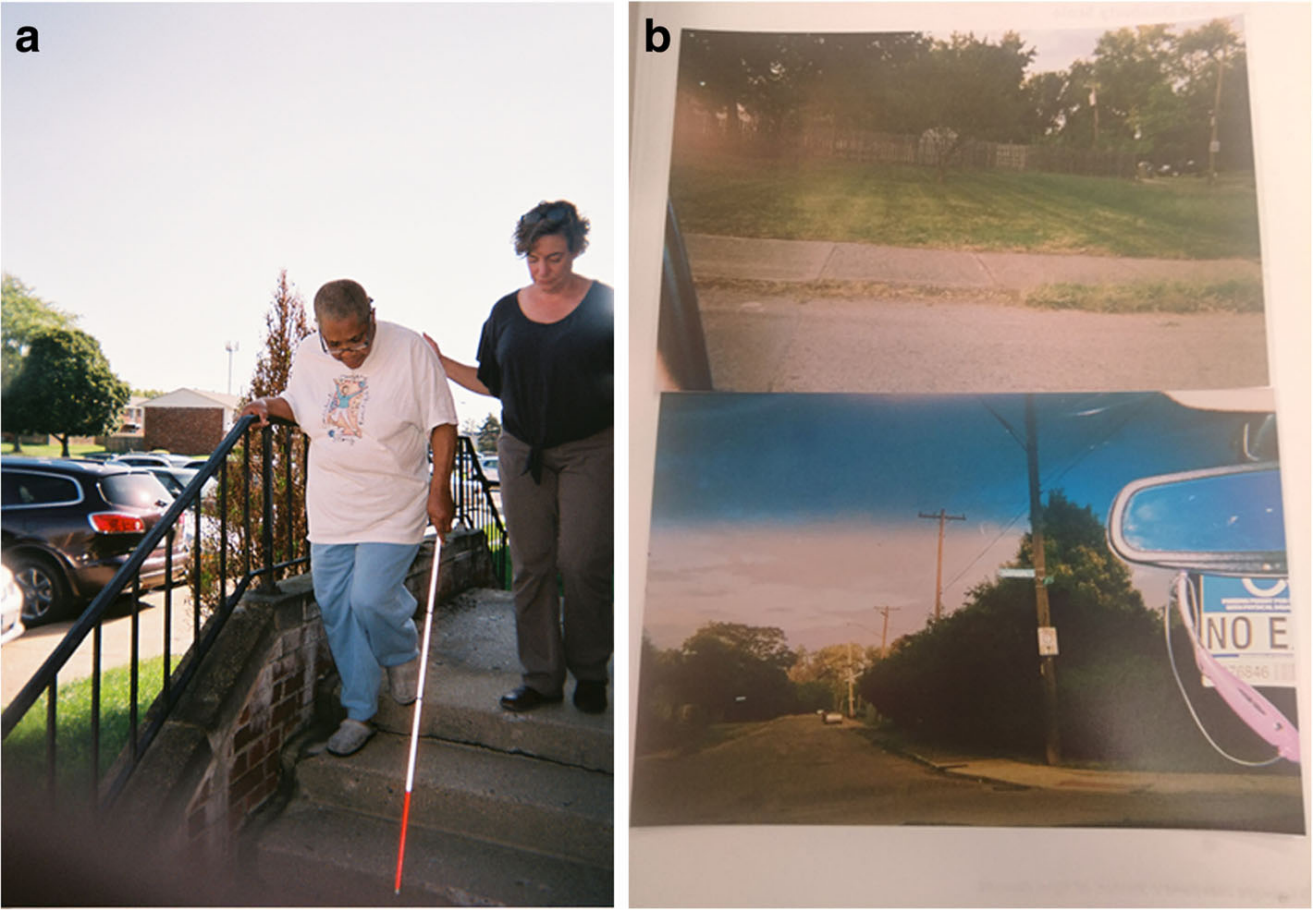
**a** and **b**: Anxiety for Latoya S. Legend 3**a**: I took a picture of my mother coming down the steps using her new stick. If you would have known my mother before she was blind in her right eye, she would get up early in the morning, you couldn’t stop her. But since this has occurred, a lot of stuff has slowed her down. She is not able to do what she used to do, and it frustrates her, and when she is frustrated she gets on my nerves. So by her learning how to use this, this is a new beginning for her, to start over, and still be independent, instead of dependent, like she does not want to be. I took the picture because she doesn’t know how proud I am of her. Between her and my kids, that’s where the majority of my anxiety comes. Because I am her home health aide, her daughter, her everything. “*That’s where the majority of my anxiety comes”.* Legend 3**b**: It used to be a house there, the house is no longer there. My brother was actually killed in that house, at the foot of my bed. And years later, somebody set it on fire, and now there is no longer that house. My great grandmother used to live there. It had a picket fence, and a line for hanging clothes. She had chickens. I can still see that house in my head. The neighbors across the street had a candy store. Now there is a pretty tree and grass there. Living there was a learning experience. I wouldn’t say happy. I learned a lot of stuff there that still stays with me today. “*I learned a lot of stuff there that still stays with me today”*

**Fig. 4:**
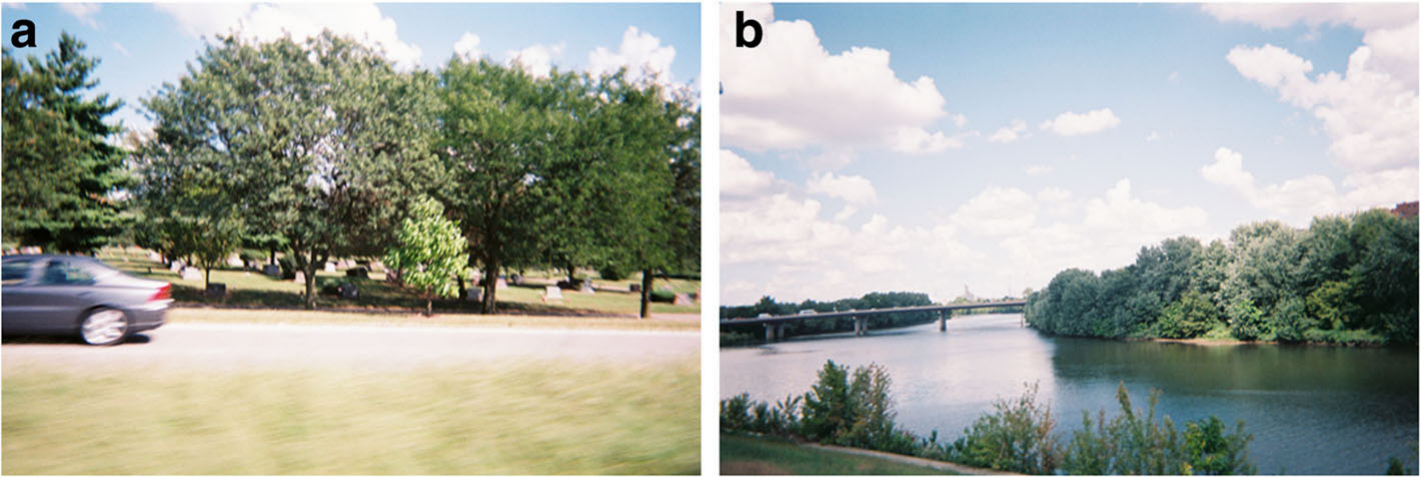
**a** and **b**: Anxiety for Andrew B. Legend 4**a**: The cemetery I am looking at it as a restful place, as it is for the dead, and also a peaceful place, they are resting in peace. And I was thinking to myself that when I have anxiety I like to be at peace, there can be so much going on, all I want is just to close the door and you want to be left alone and you want to be at peace. So that’s why I took the cemetery. “*There can be so much going on.”*Legend 4**b**: I started throwing logs that went flowing down the river. It was so beautiful, when I took that picture. My thoughts, my anxieties just floating down the river. Yeah, keeping moving. “*Just floating down the river”*

**Fig. 5:**
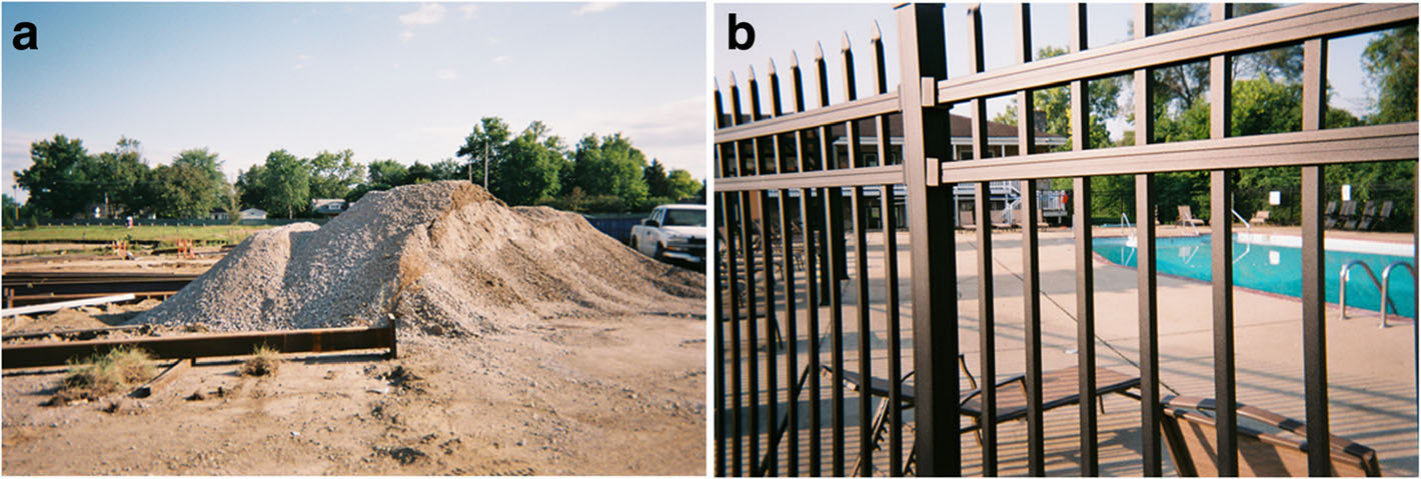
**a** and **b**: Anxiety for Chet M. Legend 5**a**: I had to go to work at a hospital. It’s a pretty big expansion. I have been there for a month. It will probably be at least another month. But compared to where we were when I started, as opposed to now, I am a Baptist, I can see the light. I took it at its current state now, and I need to be patient. Not easy for me. Like waiting in the waiting room at the ED. “*Like waiting in the waiting room at the ED”.* Legend 5**b**: I get pretty miserable in the heat. It affects my life and my anxiety, but it is hard to articulate that. I just wanted to be in that swimming pool. Like on a day like today, when I get home from work, all I want to do is get in the shower. Of course my girl, she wants to talk, but I am not in the mood for talking, I just want to relax and take it easy. All that talking makes me have anxiety. It wears on you, bad. So to me, that’s a simple picture of a swimming pool. Needless to say, that is where I want to be. “*It is hard to articulate that”*

**Fig. 6:**
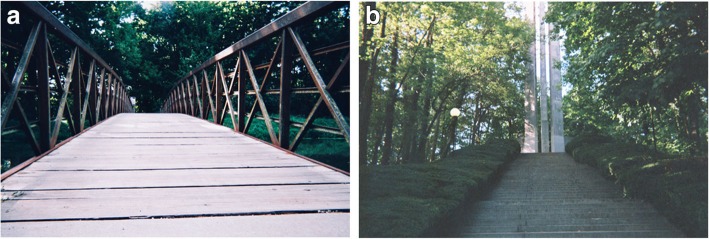
**a** and **b**: Anxiety for Alex P. Legend 6**a**: This bridge represents a connection between the state of mind that I am in when I struggle with depression and anxiety and the clarity and safety that I experience when I am calm. The water underneath the bridge mirrors the unpredictable, unwelcome, and often dangerous effects that anxiety places in my life. Although I don’t always see the water, I know it is a short distance underneath me at all times. The bridge is my barrier between anxiety and peace. To me, this safety barrier is created by my girlfriend, friends, and family who offer me guidance and love through very trying times. Additionally, I know I can rely on the strong, material structure of the bridge. Just like the planks of wood that keep me above water, physical activity, hard work, and hobbies bring me consistency, satisfaction, and power over my anxiety. Regardless of what creates the bridge, the absence of one leads to a downfall and terrifying cycle of stress. “*Anxiety has unpredictable, unwelcome, and often dangerous effects on my life”.* Legend 6**b**: For many It can be easy to dwell on the days that are particularly challenging without recognizing or appreciating the good days. This picture represents the majority of days that I have with anxiety in that there are many days where the steps in front of me look insurmountable. One of the rare benefits of anxiety that I have experienced is that the bad days with anxiety help me to really appreciative the days where I am stress free. On the good days I am at the top of these steps looking down at the beautiful view. “*I appreciate the good days a lot”*

**Fig. 7:**
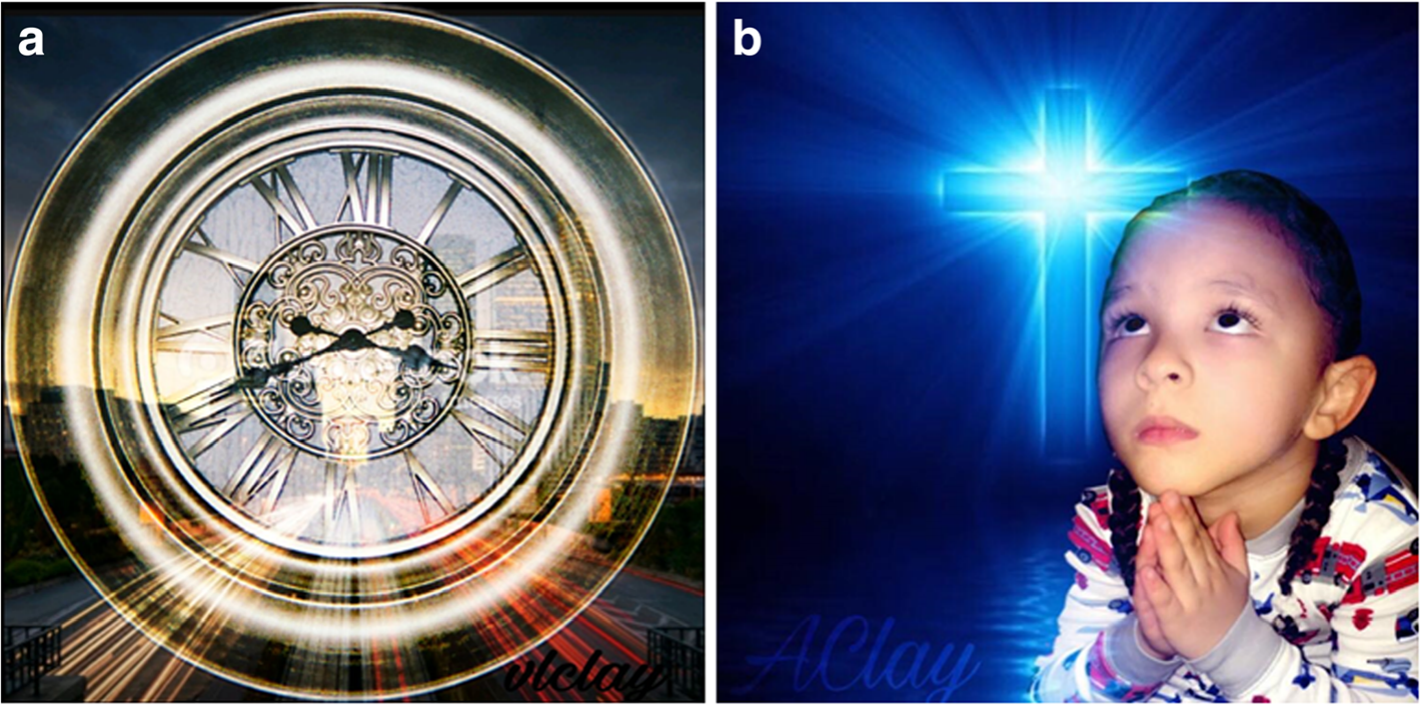
**a** and **b**: Anxiety for Veronika C. Legend 7**a**: I chose to take a picture of a clock in my home, and I added the image of the fast pace city life for the background. To me, this clock represents all the time spent dealing with anxiety, as well as time wasted trying to cope. With all the stresses brought on by the fast pace of our everyday lives, it just doesn’t seem like there is enough time in the day to get things done or even enjoy the quality of life we’d like to. I feel that a lot of our anxiety comes from the pressures of trying to keep up with the fast pace of everyday life, and sadly time just doesn’t stand still nor does life slow down to allow us to catch up! We just have to keep going and in the hustle of things, try to seek ways to help with our anxiety along the way. “*There is not enough time in the day to get things done”!.* Legend 7**b**: I had my daughter take a picture of my grandson praying. I chose this image because I feel like through our bad times as well as our good times, we should pray. I am a firm believer in prayer. I know that it is my Heavenly Father who keeps me strong and allows me to keep pressing forward everyday, and not give up when I feel I am at my weakest. Prayer has been my MAIN source of strength as well as my way to control and get through my battles with anxiety, stress and depression, especially since I prefer not to take medicine. Prayer is powerful, and I am truly a woman of faith! “*I am truly a woman of faith”!*

The final Photovoice exhibit included two posters from each participant and brief background information about anxiety, the project purpose and funder, and the Photovoice method. In addition, the content of the exhibit was also combined into a 5 × 8-in. booklet for event attendees to keep. The content of each poster, without formatting, is shown below in Figs. 2, 3, 4, 5, 6 and 7. For each figure we provide a title showing the first name and initial of the photographer (who also wrote the legends).

**Fig. 8:**
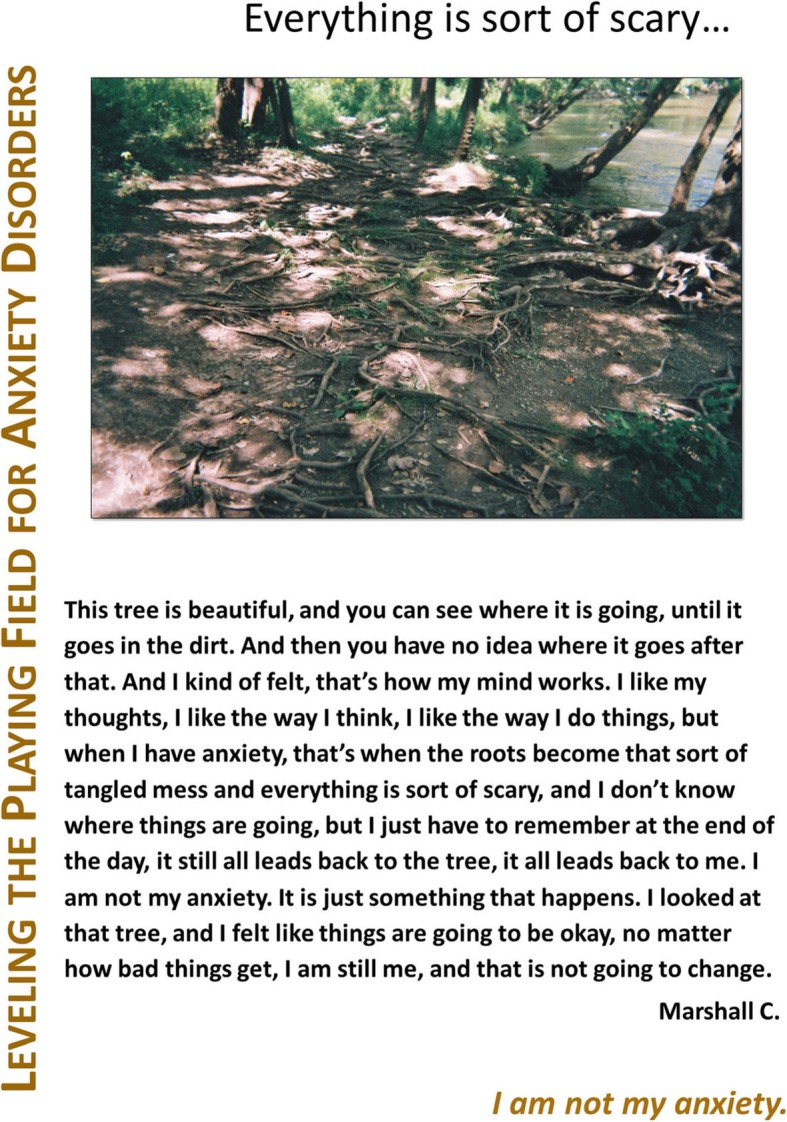
Patient Partner Poster from “Leveling the Playing Field for Anxiety Disorders”. Legend: “Leveling the Playing Field” used a format that builds on the Photovoice poster format developed by Boston University’s Center for Psychiatric Rehabilitation by adding a title and tagline [21]. Poster elements include: the exhibit title, a poster title, a participant photograph and related caption, and a tagline, intended to convey significance of experience, represented by photo and caption, from the photographer’s perspective

The project team chose “Leveling the Playing Field for Anxiety Disorders” as their exhibit title to reflect patient partners’ unique knowledge and expertise, which contributed to understanding (and empathy for) the impact of anxiety on their lives. “Leveling the Playing Field” means helping patients and providers to communicate across disciplinary boundaries, learn from each other, and collaborate to improve health and healthcare for anxiety disorders in the Emergency Department and beyond. An example of an individual poster is shown in Fig. 8.

## Discussion

The photos and captions patients chose to share in this paper reflect a wide range of perspectives on how patients think and feel about anxiety in their lives. Some pictures are concrete images that convey sources of anxiety or coping strategies whereas other pictures reflect abstract representations of what it’s like to live with an anxiety disorder from the patient’s perspective. Facilitators purposively avoided being overly-prescriptive so that patients with varying levels of awareness and ability to express their thoughts and feelings could comfortably contribute. Prior to participation, participants did not feel understood and generally thought their symptoms were minimized during care encounters in the ED. From their perspectives, the impact of anxiety on their lives was a knowledge gap that they were motivated to fill. Their photos and captions reveal the truth of their experience from their perspectives. Their photos and captions may not in fact reflect the “Truth” of anxiety disorder from a clinical perspective, as has been found with narrative-based illness research [22]. In other words, the posters reflect the raw truth of patient-partner experiences, which was effective at engaging the clinicians, researchers, and administrators who attended the wrap-up event in discussion about health and healthcare experiences with anxiety. During the wrap-up event, the Photovoice exhibit “sparked conversations that might not have happened otherwise,” according to patient partners, who also noted that attendees “were asking questions and taking notes. I was taken aback by how into it they were. They were good conversations.” Stakeholder questions involved asking about experiences of care during an Emergency Department visits, stigma, and everyday things that people go through. Patient-partners commented, “They were curious about what it is like to have anxiety,” and “They genuinely wanted to know and understand.”

Photovoice achieved its intended effects of increasing awareness among all stakeholders about the burden of anxiety in patients’ lives and the imperative of improving emergency department care for anxiety. The Photovoice results complemented epidemiological data on the research topic resulting in elevated importance of the proposed research and were instrumental in engaging broad stakeholder participation in subsequent research planning. The discussions between healthcare executives and patients contributed to pledges of support for development and submission of a research award application that included costs to the health system associated with implementing interventions to be tested. In addition, all 20 event attendees plus other identified key individuals contributed to development of the research idea during ongoing meetings. Within a two-month period, the collaborative efforts of the expanded research partnership resulted in submission of a letter of intent and invitation to apply for a research award through PCORI. Shortly thereafter, a full research proposal focused on addressing anxiety among low-risk chest pain patients in the emergency department setting was submitted and is currently under review. Our project’s focus on encouraging collaboration for future research and action is similar to other, research-based photovoice projects [23].

Participation in the Photovoice project also had a positive effect on patients, at multiple levels. On the personal level, patient-partners were able to participate in greater reflection about the impact of anxiety disorder on their lives. At a group level, Photovoice provided an opportunity to give and receive emotional support during group discussions in which they became more comfortable talking about their anxiety with others. Although the patient-partner group had been meeting together over the past 6 months under the P2P project, Photovoice participation bonded them further and created a sense of group cohesion. As noted by patient partners during a discussion dedicated to reflection on their Photovoice participation, “It led us to think outside the box and helped us to communicate about that part of our lives,” “It allowed me to open up more,” and “It helped me to dig deep within myself to better express how living with anxiety affects me by using objects and images that are all around me.” Several participants added that Photovoice was therapeutic, “It made me step back and take a look at my anxiety in ways I hadn’t before,” and “Taking pictures and reflecting on them afterwards makes you a little bit more at peace.” As such, participation in Photovoice was perceived as adding value to involvement as a patient partner for research. Photovoice also gave them an opportunity to use their lived experience to provide new and valuable information and be recognized for their contribution. One patient partner commented, “It was really nice to have others express how much they liked my photos and to even be told how great a job I had done.” The positive contribution experience carried over to an observable increase in participation during discussions about various aspects of the research plan including enrollment strategies, preferences for treatment options to be compared, and measurement of outcomes relevant to their experience of symptoms and desired functional improvements. Furthermore, the group cohesion and pride in accomplishments that developed among patient partners contributed to retention of all six patient-partners during an (unfunded) transition period following the end of the P2P engagement award and during planning and submission of the research application that included ongoing patient partner roles during conduct of the research. Empowerment and engagement observations described above support findings in reviews of photovoice in the peer-review literature [23].

### Future Improvements

Similar to other photovoice projects, we involved health system leaders as well as providers, patients, and researchers but did not formally evaluate impact of the exhibit on attitudes or perceptions of these stakeholders [23]. In addition, while seeking health equity through improved awareness of health and healthcare challenges among stakeholders (those in power) is a common goal of photovoice projects and studies [24], we did not directly measure any changes in awareness of anxiety as a serious condition. Future photovoice projects could include these issues in a formal evaluation to improve understanding of impact on all stakeholder groups.

## Conclusion

In sum, the Photovoice project component encouraged meaningful communication across all stakeholders and, along with other P2P-funded engagement activities, contributed to their increased support for the research topic and ongoing involvement in the development and submission of a research award for patient-centered outcomes research.

## Data Availability

Not applicable.

## Abbreviations

ED: Emergency department
GAD-7: Generalized Anxiety Disorder 7-item scale
P2P: Pipeline to Proposal award
PCOR: Patient-centered outcomes research
PCORI: Patient-Centered Outcomes Research Institute
